# Prevalence of Metabolic Syndrome among Korean Adolescents According to the National Cholesterol Education Program, Adult Treatment Panel III and International Diabetes Federation

**DOI:** 10.3390/nu8100588

**Published:** 2016-10-01

**Authors:** Seonho Kim, Wi-Young So

**Affiliations:** 1 Department of Nursing, Chungbuk National University, Cheongju-si, Chungbuk 28644, Korea; dipperkim@naver.com; 2 Sports and Health Care Major, College of Humanities and Arts, Korea National University of Transportation, 50 Daehak-ro, Chungju-si, Chungbuk 27469, Korea

**Keywords:** adolescent, metabolic syndrome, obesity

## Abstract

In both adults and children, metabolic syndrome (MetS) has been attributed to risk factors for type 2 diabetes and cardiovascular disease such as insulin resistance, abdominal obesity, hypertension, and dyslipidemia. This descriptive study aimed to compare the prevalence of MetS and diagnostic components according to the National Cholesterol Education Program, Adult Treatment Panel III (NCEP-ATP III) and International Diabetes Federation (IDF) in 2330 Korean adolescents (10–18 years), using data from the 2010–2012 Korea National Health and Nutrition Examination Survey-V. The NCEP-ATP III and IDF were used to diagnose MetS and yielded prevalence rates of 5.7% and 2.1%, respectively, with no sex-related differences. The most frequent MetS diagnostic components according to the NCEP-ATP III and IDF criteria were high triglyceride levels (21.2%) and low high-density lipoprotein cholesterol levels (13.6%), respectively; approximately 50.1% and 33.1% of adolescents had at least one MetS diagnostic component according to the respective criteria. Both overweight/obese male and female adolescents exhibited significantly increased prevalence rates of MetS and related diagnostic components, compared to normal-weight adolescents. In conclusion, the prevalence rates of MetS and diagnostic components differ according to the NCEP-ATP III and IDF criteria. Henceforth, efforts are needed to establish diagnostic criteria for Korean adolescents.

## 1. Introduction

In both adults and children, metabolic syndrome (MetS) has been attributed to insulin resistance, abdominal obesity, hypertension, and dyslipidemia, all of which are risk factors for type 2 diabetes and cardiovascular disease [1,2]. For example, Morrison et al. (2008) reported that children with MetS have a two-fold or three-fold higher prevalence of diabetes, which leads to increased risks of non-insulin-dependent diabetes and cardiovascular disease in adulthood [3,4,5]. Therefore, the early diagnosis and management of children with MetS are very important with regard to public health [6,7,8].

Notably, the rapid development of MetS characteristics in children can make it difficult to determine the cutoff values of diagnostic components for MetS [9]. Large changes in triglyceride and cholesterol levels occur during adolescence, and age-related increases in both blood pressure and body mass index (BMI) have been associated with characteristics of physiological insulin resistance. Accordingly, no common criteria for MetS are currently in use [9], although for children, most researchers adopt or modify the criteria of the National Cholesterol Education Program, Adult Treatment Panel III (NCEP-ATP III) and the International Diabetes Federation (IDF).

Previous studies have reported the prevalence of MetS in children according to the above-mentioned criteria, age, race, and degree of obesity; in particular, prevalence rates were found to differ significantly according to the criteria [10]. In Korean studies of children, the prevalence rates of MetS according to the modified NCEP-ATP III [6,11,12,13] and IDF criteria [10,13,14,15] ranged from 3.8% to 9.7% and from 1.0% to 4.2%, respectively.

Obesity is an important factor in pediatric MetS, and previous studies have reported a higher prevalence of MetS among obese children when compared to their normal-weight peers [10,11,12,16,17,18]. Because pediatric obesity is known to extend into adulthood [19], it is essential to manage this condition in order to prevent the development of MetS in adulthood.

According to reports, the prevalence of MetS is decreasing among Korean children [6,10,13]. Yoon [13] reported that the prevalence of MetS in children tended to increase from 1998 to 2001, but later decreased from 2005 to 2007, whereas Lee et al. [6] reported that the prevalence of pediatric MetS decreased between 1998 and 2008. Furthermore, Jin et al. [10] recently reported that the prevalence of pediatric MetS decreased between 2007 and 2010.

In most studies of Korean children, the prevalence of MetS was determined using data collected before 2008, and no reports have addressed recent prevalence rates. To address this lack of information, the principal objective of this study was to compare the prevalence of MetS among Korean adolescents according to the NCEP-ATP III and IDF definitions, using data from the 2010–2012 Korea National Health and Nutrition Examination Survey-V (KNHANES-V). The secondary objectives were to compare the diagnostic components in different strata based on sex or BMI categories.

## 2. Methods

### 2.1. Original Data and Samples

We used raw data from the KNHANES-V (2010–2012), a study conducted by the Korean Center for Disease Control (KCDC). The KNHANES is a sample survey in three sections (examination, health interview, and nutritional survey) that targets the entire national population. After excluding subjects with incomplete data, 2330 children aged 10–18 years were included in this study. All participants provided informed consent before data collection, and the survey was approved by the KCDC Bioethics Committee (approval numbers: 2010-02CON-21-C, 2011-02CON-06-C, and 2012-01EXP-01-2C in 2010, 2011, and 2012, respectively).

### 2.2. Measures

Anthropometry, blood pressure measurement, and blood analysis protocols are well-described in the KNHANES-V guidelines [20]. Anthropometry was conducted by an expert inspector, who measured weight to the nearest 0.1 kg using a scale (GL-6000-20, Seoul, Korea) and height to the nearest 0.1 cm using an extensometer (Seca 225; Seca GmbH, Hamburg, Germany). Waist circumference was measured to the nearest 0.1 cm at the midpoint between the lowest ribs and the iliac crest during exhalation. The BMI was calculated by dividing the weight (kg) by the height (m) squared; subjects were classified as normal weight, overweight, or obese when the BMI fell into the <85th, 85th–94th, or >95th percentile or 25 kg/m^2^, using the 2007 growth diagram suggested by the KCDC and Association of Korean Pediatrics [21]. Blood pressure was measured three times with a mercury sphygmomanometer (Baumanometer^®^ desk model 0320; WA Baum, Co., Copiague, NY, USA) after an appropriate tourniquet was applied to the right arm and the subject rested in a seated position for 5 minutes; the average of the second and third measurements was ultimately recorded as the blood pressure.

Blood was collected after at least 8 h of fasting and then stored in a refrigerator. Blood samples were examined within 24 h after transfer to the examination center. Plasma triglyceride, high-density lipoprotein cholesterol (HDL-C), and fasting glucose levels were analyzed in plasma using an automatic analyzer (ADVIA 1650; Siemens, New York, NY, USA).

### 2.3. Definition of MetS

In this study, MetS was defined using the modified NCEP-ATP III and 2007 IDF criteria according to age (children), as suggested by Ford et al. [17] and the IDF [22], respectively. Regarding diagnostic criteria, the abdominal obesity criteria incorporated waist circumference, as suggested by the Korean Society for the Study of Obesity in 2005 as appropriate for a Korean population [23], and the 2007 Korean national growth chart for children and adolescents [21]. The modified NCEP-ATP III and IDF criteria each contained five parameters: Waist circumference, and the levels of triglyceride, HDL-C, fasting blood glucose, and blood pressure; however, these criteria differed with regard to standard values and MetS diagnostic criteria. According to the modified NCEP-ATP III, three of the five criteria must be satisfied for a diagnosis of MetS. However, abdominal obesity is considered an essential factor for MetS diagnosis according to the IDF, and therefore in this study, MetS was diagnosed when three of the five criteria, including waist circumference, were satisfied (Table 1).

### 2.4. Statistical Analysis

Participants’ anthropometry values and blood levels were expressed as means and standard deviations; independent *t*-tests were used for comparisons between the sexes. The chi-square test and linear trends chi-square test were used to examine differences in MetS and diagnostic components according to sex and obesity status. The data analysis was conducted using SPSS version 20.0 (SPSS, Chicago, IL, USA). A *p*-value < 0.05 was used to define statistical significance.

## 3. Results

Comparisons of anthropometric values and blood levels according to sex are shown in Table 2. Boys had a significantly greater height, weight, waist circumference, BMI, systolic blood pressure, and fasting glucose value when compared to girls (*p* < 0.05). In contrast, girls had greater HDL-C (*p* < 0.01) and triglyceride levels (*p* = 0.001). No significant sex-related differences were observed in age and diastolic blood pressure (*p* > 0.05).

The MetS prevalence according to the modified NCEP-ATP III criteria was 5.7%; this rate was 3.6% higher than the rate of 2.1% determined using the IDF criteria. The prevalence of four diagnostic components (except low HDL-C) was higher when the modified NCEP-ATP criteria were used (Table 3). According to the modified NCEP-ATP III criteria, the distribution of MetS diagnostic components was as follows (in decreasing order): high triglyceride (21.2%), elevated blood pressure (20.4%), low HDL-C (11.6%), elevated fasting glucose (11.4%), and abdominal obesity (9.7%); 50.1% of the children had more than one diagnostic component. In contrast, the IDF criteria yielded a different distribution of MetS diagnostic components (in decreasing order): Low HDL-C (13.6%), elevated fasting glucose (11.4%), abdominal obesity (9.4%), high triglyceride (7.8%), and elevated blood pressure (2.4%); here, only 33.1% of children had more than one diagnostic component. Among the IDF criteria, only elevated blood pressure differed significantly according to sex (*p* = 0.001).

The prevalence trends of MetS and the diagnostic components according to obesity status are shown in Table 4. As the weight status increased from normal to obese, the prevalence of MetS tended to increase when the modified NCEP-ATP III criteria were adopted, compared with the IDF criteria. According to the modified NCEP-ATP III criteria, overweight or obese children of both sexes exhibited significantly increased prevalence rates of MetS and diagnostic components (*p* < 0.01), although elevated blood pressure was not significant in girls (*p* = 0.541). According to the IDF criteria, all diagnostic components, except blood pressure in girls (*p* = 0.486), as well as the prevalence of MetS, were increased in overweight and obese participants (*p* < 0.01).

## 4. Discussion

In this study, we analyzed the prevalence of MetS and related diagnostic components among Korean adolescents, using the widely accepted modified NCEP-ATP III criteria and IDF criteria and data representative of the Korean national population. According to our results, the prevalence rates of MetS among Korean children aged 10–18 years were 5.7% and 2.1%, based on the modified NCEP-ATP III and IDF criteria, respectively. These results were similar to the reported prevalence rates of MetS among children in previous advanced studies [6,10,11,12,13,14,15]; however, it is difficult to directly compare our findings with those of earlier studies because studies conducted prior to 2008 used different criteria. We note that in a previous study based on the modified NCEP-ATP III criteria, the MetS prevalence in Korea was found to decrease from 7.6% in 1998 to 3.8% in 2008 [6]. Furthermore, Jin et al. [10] applied the IDF criteria to determine that the MetS prevalence decreased from 3.1% in 2007 to 1.0% in 2010 in Korea. A review of several Korean studies [6,10,11,12,13,14,15] revealed that the prevalence of MetS increased up to the early 2000s and began to decrease after the late 2000s. Given the above-described challenges regarding direct comparisons of the MetS prevalence, the trends of MetS and diagnostic components among Korean children must be determined over time. Accordingly, additional studies that aim to understand trends in the prevalence of MetS among Korean children will likely use the modified NCEP-ATP III and IDF criteria.

In this study, the modified NCEP-ATP III criteria yielded a higher prevalence of MetS, compared to the results obtained with the IDF criteria. This difference might be attributable to the use of different criteria and diagnostic component cut-off points. The IDF and modified NCEP-ATP III criteria differ because the former regard abdominal obesity as an essential factor, a restriction that negatively affected the prevalence of MetS in this study. Furthermore, the modified NCEP-ATP III considers sex, age, and height and uses percentiles to diagnose elevated blood pressure and other cutoff points; for example, the cutoff triglyceride level in this set of criteria was 110 mg/dL, which corresponds to the 90th percentile, rather than 150 mg/dL, which is used in the criteria for adults. We observed large differences in the frequencies of elevated blood pressure (20.4% and 2.4%, respectively) and triglyceride levels (21.2% and 7.8%, respectively) when comparing results obtained with the modified NCEP-ATP III and IDF criteria. However, evidence supporting changes in the reference values (i.e., modified NCEP-ATP III and IDF criteria) for children remains unclear, and further studies regarding the pediatric cut-off points for these characteristics are needed. Furthermore, inter-comparisons and analyses of the prevalence of MetS in children should be conducted with caution, as the prevalence of MetS has been shown to vary according to the diagnostic criteria.

The distribution of MetS diagnostic components in this study agreed with those of advanced studies in which high triglyceride, elevated blood pressure, and low HDL-C levels were found to be the strongest diagnostic components for MetS, according to the modified NCEP-ATP III [11,12]. Other studies that implemented the IDF criteria found that a low HDL-C level was the strongest diagnostic component [10,13,14,15]. These differences in distribution might be attributable to the use of different standards for measuring blood pressure, triglyceride, and HDL-C levels.

In our study, approximately 50.1% and 33.1% of children were found to have more than one diagnostic component for MetS when using the modified NCEP-ATP III and IDF criteria, respectively. Furthermore, one out of every two to three Korean children was found to have a MetS diagnostic component. Pediatric metabolic abnormalities in children can easily be transferred to adulthood [7], and children with multiple diagnostic components have a greater risk of developing another metabolic abnormality [24]; therefore, it is very important to diagnose and manage MetS at an early stage. In addition, the early identification of MetS diagnostic components is an important step with regard to treatment decisions and disease prevention [7]. Future studies should address effective screening for the early identification of MetS and preventive interventions.

In this study, the prevalence of MetS was higher among boys (5.8%) than among girls (5.5%) according to the modified NCEP-ATP III, but higher among girls (2.2%) than among boys (1.9%) according to the IDF criteria, despite a lack of significant differences. In previous studies, boys generally had a higher prevalence of MetS [12,25,26], although some researchers [11,12] have reported a lack of significant sex-based differences [11,12] and inconsistent findings. Among the diagnostic components used in this study, only elevated blood pressure, from both the modified NCEP-ATP III and IDF criteria, was significantly higher in boys than in girls. This difference is attributed to the fact that boys and girls exhibited a similar prevalence according to the modified NCEP-ATP III criteria, using which elevated blood pressure is diagnosed when values exceed the 90th percentile for age and height in adolescents younger than 18 years, whereas the IDF criteria incorporate an absolute value of 130/85. A similar study of sex-based differences in the prevalence of MetS and diagnostic components among Korean children is likely needed in future.

Generally, obese children have a higher prevalence of MetS when compared to children with a normal BMI [11,12,16,17,18]. In the present study, the frequencies of MetS among children with normal BMI values were 1.7% and 0.4% according to the modified NCEP-ATP III and IDF criteria, respectively. For comparison, the corresponding MetS frequencies among obese children were 29.7% and 0.4%, respectively, and these findings corroborate previous studies in which a higher prevalence of MetS was observed in obese children [11,17,18]. Obesity plays a central role in the development of MetS [10,17,27,28] and, in particular, pediatric obesity is linked to adult obesity and serves as a predictive factor for MetS [28]. Sun et al. [28] reported that people with systemic and abdominal obesity during childhood had a 2.8-fold higher risk of developing MetS at an age >30 years, and this risk correlated significantly with an obese status beyond the age of eight years. Among Korean adolescents, the obesity rate remained stagnant until 2010 but began to increase in 2011 [29]. Accordingly, the prevalence of MetS is expected to increase unless obesity is prevented and managed. Furthermore, obesity management is very important because 65% of overweight and 82% of obese children are expected to develop adult obesity [19].

Worldwide, as in Korea, there is currently no consensus regarding the criteria for MetS in children and adolescents. Similarly, consensus has not been reached regarding whether the NCEP-ATP III and IDF criteria are more appropriate for Korean adolescents. To address this problem, definitive longitudinal studies that compare the abilities of the IDF and NCEP criteria to predict the risk of adulthood MetS are necessary.

This study had several limitations. First, this was a cross-sectional study, despite the use of national health and nutrition survey data representative of the national population. Because our study was limited to identifying trends in MetS prevalence according to sex and weight status, a longitudinal study will be needed in the future. Second, we did not consider several factors that could affect MetS. For example, children experience rapid growth and development with age, and adolescence marks a period of insulin resistance that leads to abnormal glucose and fat metabolism. Although the adolescent developmental status should have been considered, no relative survey was conducted to obtain this data. However, despite these limitations, the strengths of our study include the use of national representative health and nutrition survey data and an investigation of the sex- and weight-based prevalence of MetS in Korean children using two criteria that have been recognized worldwide.

## 5. Conclusions

The prevalence of MetS among Korean children and adolescents remains high, and one of every two or three subjects in our study had more than one metabolic syndrome diagnostic component. Although we did not observe a sex-based difference in the MetS prevalence, overweight and obese adolescents exhibited a high prevalence relative to their normal-weight counterparts. A subsequent large longitudinal study to establish appropriate diagnostic criteria for Korean adolescents is needed.

## Figures and Tables

**Table 1:** Metabolic syndrome diagnostic criteria for an adolescent population.

Criteria	NCEP-ATP III	IDF
Diagnostic Criteria	Three of more of the following	Abdominal obesity and two other criteria
Waist circumference	≥90th percentile for age and gender	≥90 percentile for age and gender (<16 yr)
		≥90 cm for male, ≥85 cm for female (≥16 yr)
Fasting glucose	≥110 mg/dL	≥100 mg/dL or diabetes
Blood pressure	≥90th percentile for age, gender and height (<18 yr) ≥130/85 mmHg (≥18 yr)	≥130/85 mmHg (<16 yr)
		≥130/85 mmHg or drug treatment for
		hypertension (≥16 yr)
Triglycerides	≥110 mg/dL	≥150 mg/dL (<16 yr)
		≥150 mg/dL or drug treatment for elevated
		triglycerides (≥16 yr)
HDL-C	<40 mg/dL	<40 mg/dL (<16 yr)
		<40 mg/dL for male, <50 mg/dL for female or drug treatment for low HDL-C (≥16 yr)

NCEP-ATP III, National Cholesterol Education Program, Adult Treatment Panel III; IDF, International Diabetes Federation; yr, Years; HDL-C High-density lipoprotein cholesterol.

**Table 2:** Anthropometric and metabolic characteristics of the subjects by sex.

Variables	Boys (*n* = 1249)	Girls (*n* = 1081)	*p*
Anthropometric characteristics			
Age (years)	13.7 ± 2.5	13.7 ± 2.5	0.883
Height (cm)	163.2 ± 12.8	156.6 ± 8.3	<0.001
Weight (kg)	56.1 ± 15.3	49.7 ± 11.0	<0.001
Body mass index (kg/m^2^)	20.7 ± 3.8	20.1 ± 3.4	<0.001
Metabolic characteristics			
Waist circumference (cm)	70.6 ± 10.3	66.5 ± 8.3	<0.001
Systolic blood pressure (mmHg)	107.0 ± 11.0	103.0 ± 9.3	<0.001
Diastolic blood pressure (mmHg)	64.8 ± 10.1	64.7 ± 8.4	0.744
Triglycerides (mg/dL)	79.7 ± 43.8	86.0 ± 51.5	0.001
High-density lipoprotein cholesterol (mg/dL)	52.8 ± 10.5	55.3 ± 10.8	<0.001
Fasting glucose (mg/dL)	89.6 ± 7.9	88.9 ± 9.8	0.047

*p*-values are presented as means ± standard deviations; Data were analyzed using independent *t*-tests.

**Table 3:** Distribution of metabolic syndrome and diagnostic component frequencies.

	Total		Modified NCEP-ATP III			IDF		
	Modified NCEP-ATP III	IDF	Boys	Girls	*p*	Boys	Girls	*p*
Metabolic syndrome (%)	5.7	2.1	5.8	5.5	0.687	1.9	2.2	0.613
Diagnostic components								
Abdominal obesity (%)	9.7	9.4	8.6	11.0	0.055	9.5	9.2	0.760
Elevated blood pressure (%)	20.4	2.4	20.2	20.6	0.787	3.4	1.2	0.001
High triglycerides (%)	21.2	7.8	20.3	22.3	0.249	7.4	8.3	0.389
Low HDL-C (%)	11.6	13.6	12.7	10.3	0.064	12.7	14.7	0.165
Elevated fasting glucose (%)	11.4	11.4	12.2	10.4	0.152	12.2	10.4	0.152
No. of diagnostic components (%)								
1	32.6	23.7	32.0	33.2	0.912	23.7	23.7	0.748
2	11.8	7.3	11.7	11.8		7.8	6.8	
3	4.6	1.8	4.6	4.5		1.7	1.9	
4	0.9	0.3	1.2	0.6		0.2	0.4	
5	0.2	0.0	0.0	0.4		0.0	0.0	

NCEP-ATP III, National Cholesterol Education Program, Adult Treatment Panel III; IDF, International Diabetes Federation; HDL-C, high-density lipoprotein cholesterol; Data were analyzed using the chi-square test.

**Table 4:** Distributions of metabolic syndrome and diagnostic component frequencies by body mass index.

Classification	MetS		Modified NCEP-ATP III					IDF				
	Modified NCEP-ATP III	IDF	Diagnostic Components					Diagnostic Components				
			Abd Obesity	High TG	Elevated BP	Low HDL-C	Elevated FG	Abd Obesity	High TG	Elevated BP	Low HDL-C	Elevated FG
Total												
Normal (*n* = 1,906)	1.7	0.4	0.9	16.9	19.2	8.8	9.9	0.7	5.3	1.6	10.5	9.9
Overweight (*n* = 161)	13.0	5.6	28.4	37.9	17.4	16.1	18.0	30.4	16.1	2.5	17.4	18.0
Obese (*n* = 263)	29.7	12.2	64.6	42.6	31.2	29.3	17.9	59.3	20.9	7.6	34.2	17.9
*p*	<0.001	<0.001	<0.001	<0.001	<0.001	<0.001	<0.001	<0.001	<0.001	<0.001	<0.001	<0.001
Boys												
Normal (*n* = 1,014)	1.7	0.3	0.3	15.3	17.9	9.9	10.8	0.4	4.8	2.1	9.9	10.8
Overweight (*n* = 71)	9.9	4.2	15.5	47.9	16.9	14.1	18.3	29.6	19.7	4.2	14.1	18.3
Obese (*n* = 164)	29.9	11.0	57.3	39.6	35.4	29.9	18.3	57.3	17.7	11.0	29.9	18.3
*p*	<0.001	<0.001	<0.001	<0.001	<0.001	<0.001	0.003	<0.001	<0.001	<0.001	<0.001	0.003
Girls												
Normal (*n* = 892)	1.8	0.4	1.6	18.7	20.5	7.5	8.9	1.0	5.8	1.1	11.2	8.9
Overweight (*n* = 90)	15.6	6.7	32.2	30.0	17.8	17.8	17.8	31.1	13.3	1.1	20.0	17.8
Obese (*n* = 99)	29.3	14.1	76.8	47.5	24.2	28.3	17.2	62.6	26.3	2.0	41.4	17.2
*p*	<0.001	<0.001	<0.001	<0.001	0.541	<0.001	0.001	<0.001	<0.001	0.486	<0.001	0.001

MetS, Metabolic syndrome; NCEP-ATP III, National Cholesterol Education Program, Adult Treatment Panel III; IDF, International Diabetes Federation; Abd, Abdominal; TG, Triglycerides; BP, Blood pressure; HDL-C, High-density lipoprotein cholesterol; FG, Fasting glucose; Data were analyzed using the linear trends chi-square test.
